# Portal vein embolization following arterial portography for the management of an active portal bleeding after blunt liver trauma in a cirrhotic patient

**DOI:** 10.1186/s42155-023-00423-5

**Published:** 2024-01-04

**Authors:** Romain L’Huillier, Bénédicte Cayot, Jean Turc, Laurent Milot

**Affiliations:** 1 Department of Diagnostic and Interventional Radiology, Hôpital Edouard Herriot, Hospices Civils de Lyon, University of Lyon, Pavillon B, 5 Place D’Arsonval, Lyon, 69003 France; 2 LabTAU - INSERM U1032, Lyon, 69003 France; 3 The French Comprehensive Liver Center, Hospices Civils de Lyon, University of Lyon, Lyon, 69004 France; 4 Department of Anesthesiology and Intensive Care Unit, Hôpital Edouard Herriot, Hospices Civils de Lyon, University of Lyon, Lyon, 69003 France

**Keywords:** Cirrhosis, Portal vein embolization, Arterial portography, Liver trauma

## Abstract

**Background:**

The management of blunt liver trauma in cirrhotic patients is challenging, because while bleeding is most often of arterial origin, the increased pressure in the portal system associated with cirrhosis can increase the risk of portal bleeding, which is sometimes difficult to confirm on contrast-enhanced abdominal computed tomography.

**Case presentation:**

We managed a 54-year-old cirrhotic patient who presented with blunt liver trauma. Computed Tomography showed active intraperitoneal bleeding presumed to be of hepatic origin. Given the patient's hemodynamic stability, the decision was made to manage the patient non-surgically. The patient underwent hepatic arteriography to rule out an arterial origin to the bleeding. A superior mesenteric arterial portography confirmed the portal venous origin of the bleeding. To stop the bleeding, a distal portal vein embolization using coils and glue was performed by approaching a large paraumbilical vein.

**Conclusions:**

Our case study shows the value of arterial portography in the management of these patients, when they are clinically stable enough to benefit from non-surgical management; This allows arterial bleeding to be excluded on hepatic arteriography, portal bleeding to be confirmed on portography following arteriography in the superior mesenteric artery, and guidance of portal vein embolization.

## Background

Liver is the most frequent site of injury in patients with abdominal blunt trauma [1]. The treatment of blunt liver trauma has changed over the last few decades: today, the majority of patients benefit from non-operative management [2]. The choice of operative or non-operative management mostly depends on liver injury grade, hemodynamic status and associated injuries [3].

Arterial lesions are the most frequent [4] and the presence of active arterial bleeding or an arterial pseudoaneurysm at the initial phase justifies arterial embolization in the absence of hemodynamic instability [3]. Portal venous bleeding is rare, because portal pressure is usually low. When severe enough to require urgent management, portal venous lesions are often associated with injuries to adjacent organs, requiring surgical management [5].

This case describes the non-surgical management of a blunt liver trauma with active intra-peritoneal bleeding of portal origin in a cirrhotic patient with portal hypertension, identified on arterial portography after a negative hepatic arteriography leading to the successful percutaneous embolization of the portal venous bleeder.

## Case presentation

A 54-year-old patient with a history of hepatic cirrhosis and known portal hypertension (porto-systemic gradient measured at 17 mmHg during a transjugular liver biopsy performed 6 months prior) was transferred to the trauma center at the Edouard Herriot University Hospital (Hospices Civils de Lyon, France) for injuries sustained after a scooter accident. His initial heart rate was 85 bpm and blood pressure was 100/73 mmHg. Hemoglobin was 9,6 g/dL and platelets count was 68,000 /µL.

**Fig. 1:**
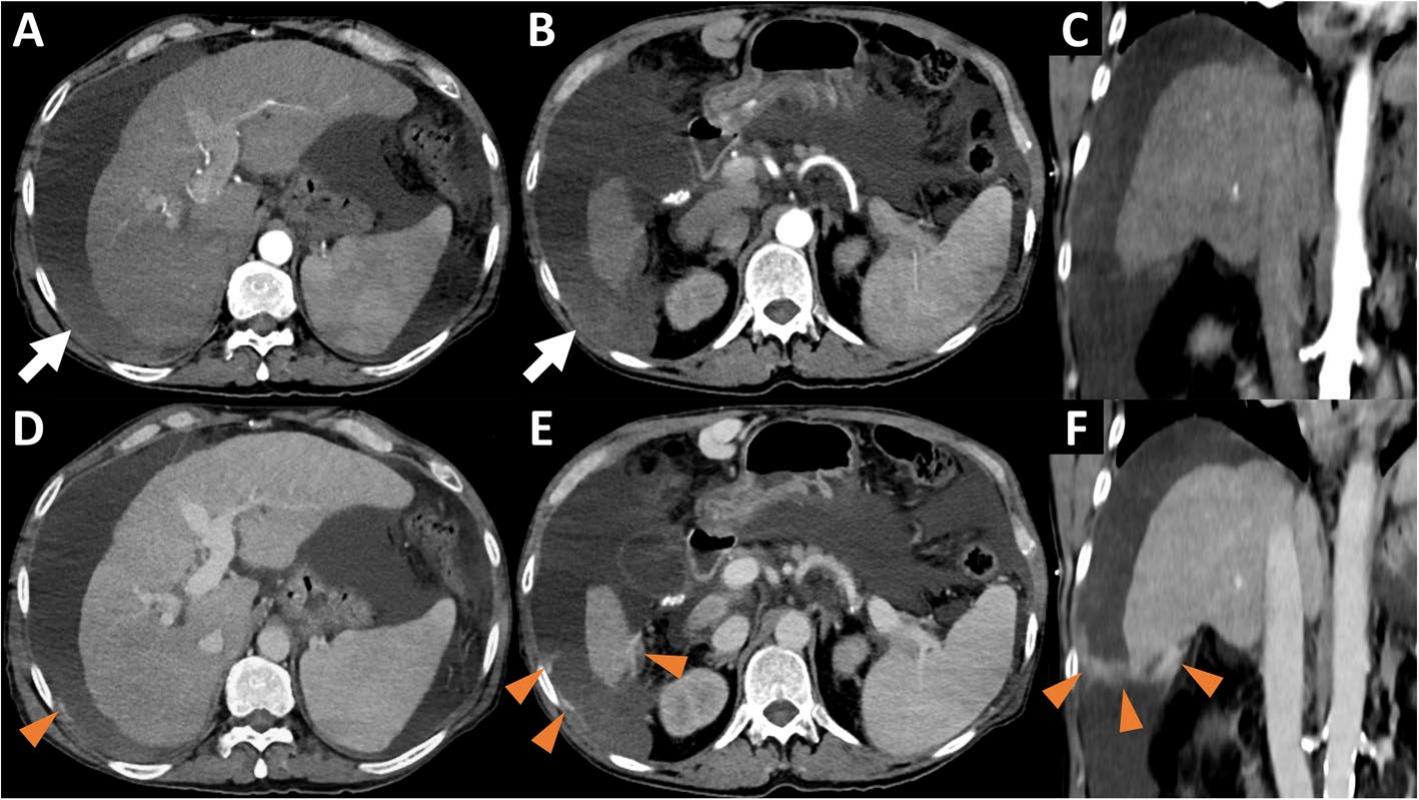
Pre-interventionnal Computed Tomography. Arterial phase in axial (**A**, **B**) and coronal (**C**) sections and portal phase in axial (**D**, **E**) and coronal (**F**) sections showing liver cirrhosis with a large intraperitoneal effusion, with perihepatic hyperdensity (white arrows) testifying to hemoperitoneum, without active bleeding in the arterial phase but with extravasation of contrast agent in the portal phase (orange arrowheads)

Contrast-enhanced abdominal computed tomography (CT) was performed in order to assess traumatic injuries and showed liver cirrhosis with signs of marked portal hypertension (splenomegaly, porto-systemic shunts) and perihepatic hemoperitoneum, associated with active intraperitoneal perihepatic bleeding adjacent to segment VI, visualized at the portal phase (Fig. 1).

As the patient was clinically stable, non-surgical management of this active intraperitoneal bleed presumed to be of hepatic origin was preferred. The patient was then transferred to the interventional radiology suit.

**Fig. 2:**
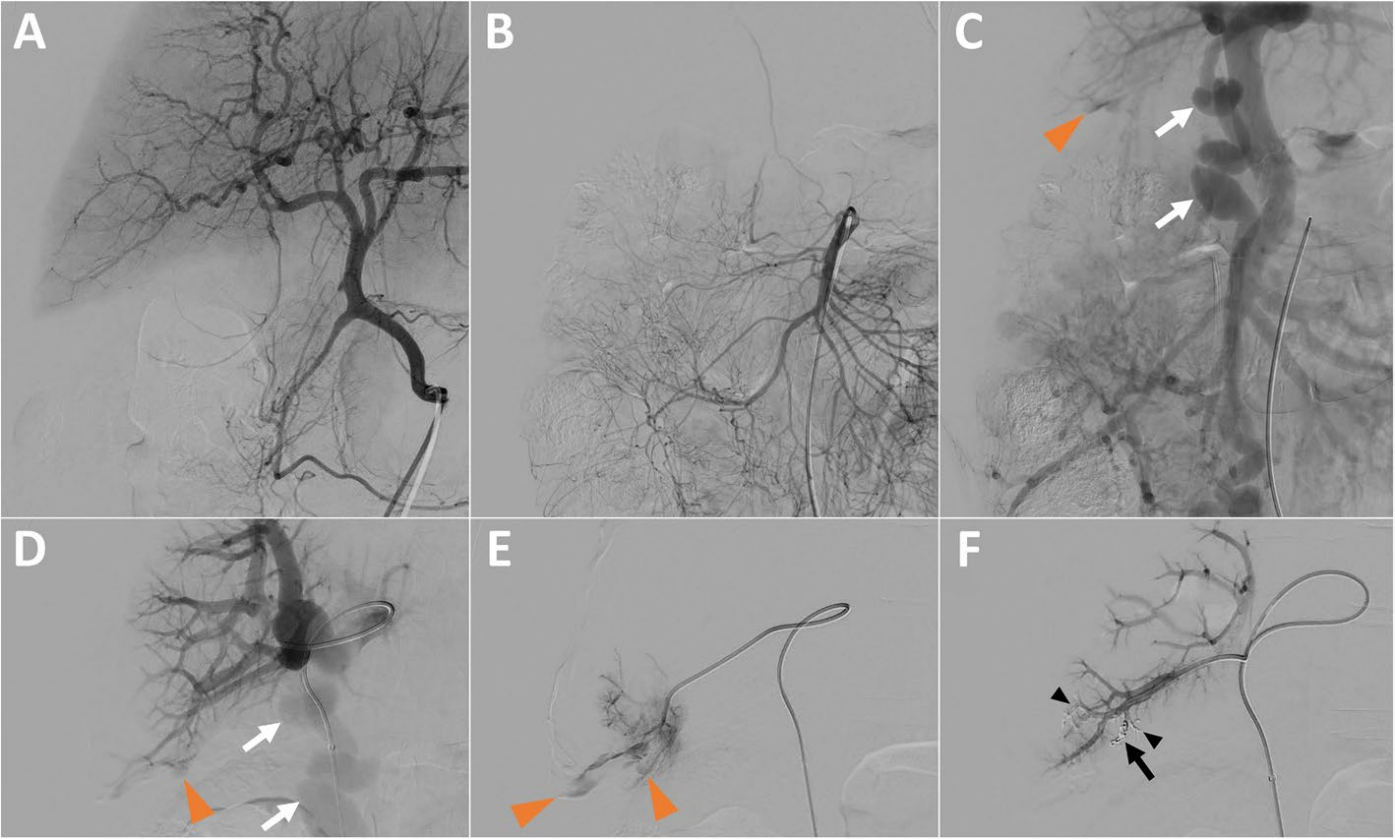
Portal Vein Embolization (PVE) after hepatic arteriography, guided by arterial portography. **A** Hepatic arteriography showing no arterial active bleeding. **B** & **C** Superior mesenteric arteriography (**B**) with subsequent arterial portography (**C**) showing active intraperitoneal bleeding of portal origin in segment VI (orange arrowhead) and a voluminous paraumbilical vein (white arrows). **D** Direct portography after ultrasound-guided puncture of the paraumbilical vein (white arrows) showing active portal bleeding in segment VI (orange arrowhead). **E** Catheterization of a distal portal branch in segment VI and injection of contrast agent to confirm the location of the bleeding (orange arrowheads). **F** Control portography, after embolization with 2 detachable microcoils (black arrow) and a glue/lipidol mixture (black arrowheads), confirming complete cessation of bleeding

Hepatic angiography revealed no active arterial bleeding, pseudoaneurysm or arteriovenous fistula (Fig. 2A).

Arterial portography following angiography of the superior mesenteric artery revealed intraperitoneal bleeding related to a distal portal venous injury in segment VI and also showed a large paraumbilical vein (Fig. 2B & C).

Under ultrasound guidance, a 5-french sheath was introduced in the paraumbilical vein, in order to avoid a transhepatic approach which is associated with a higher risk of bleeding. Direct portography confirmed active venous bleeding in segment VI, with intraperitoneal extravasation of contrast agent (Fig. 2D & E). Venous bleeding was controlled by embolization using 2 detachable 4 mm diameter micro-coils (Concerto Helix; Medtronic, Minneapolis, MN, USA) and a mixture of glue (Glubran 2; GEM, Viareggio, Italy) and lipiodol (Lipiodol Ultra-Fluid; Guerbet, Villepinte, France). Post embolization portography confirmed that the intraperitoneal bleeding had stopped (Fig. 2F). The vascular access in the paraumbilical vein was managed by manual compression during 10 min.

The day after distal portal vein embolization patient’s aspartate aminotransferase/alanine aminotransferase rose at 486/101 U/L from a baseline at 74/30 U/L. The patient remained hemodynamically stable for 24 h. Unfortunately, the progression of an intra-parenchymal cerebral hematoma led to his death 24 h after embolization.

## Discussion

For decades, the standard of care of blunt liver trauma for hemodynamically stable patients has gradually shifted from surgical to non-operative management. Surgical treatment, which used to be standard of care, remains sometimes indicated in unstable patients, unresponsive to fluid resuscitation and non-operative management.

The increasing use of arteriography and hepatic embolization contributes to the development of non-surgical management of blunt liver trauma, even in cases of active bleeding [6]. The hepatic arterial system is the most frequent source of hemorrhage from blunt liver trauma [7]. Bleeding from portal venous system is rare, because of its low pressure [8]. In cirrhotic patients with blunt liver trauma, portal hypertension, coagulopathy, and varices may increase the risk of portal bleeding [9]. In hemodynamically stable patients, a multiphasic abdominal CT is usually helpful in identifying the venous origin of the bleeding, as seen in this clinical situation.

The management of blunt liver trauma with portal bleeding in cirrhotic patients is challenging: some teams propose surgical management (packing, portal vein suture or ligature) [8], while others have proposed Transjugular Intrahepatic Portosystemic Shunt (TIPS) with or without associated portal vein embolization; or trans porto-systemic shunts embolization [10].

Portal vein embolization for portal vein injury after blunt trauma has already been described [11]. In our case, we wanted to exclude an arterial origin to the hepatic bleeding and to safely have a portography; we subsequently performed an arterial portography first. Once the portal bleeding had been identified and localized, we performed an ultrasound-guided approach of the paraumbilical vein to avoid an at risk hepatic puncture in this patient (ascites, coagulopathy), allowing us to embolize the injured distal portal branch responsible for the bleeding. We did not opt for TIPS, given the easier and quicker access to the portal system via the para-umbilical vein.

## Conclusion

Our report demonstrates the value of performing an arterial portography after negative hepatic arteriography in clinically stable patients presenting a hepatic trauma with active bleeding, in order to detect and treat bleeding of portal origin, especially in the presence of risk factors of failure of conservative management.

## Data Availability

The datasets used during the current study are available from the corresponding author on reasonable request.

## Abbreviations

CT: Computed Tomography
TIPS: Transjugular Intrahepatic Portosystemic Shunt
PVE: Portal Vein Embolization
